# Histomorphometric evaluation of seminiferous tubules and stereological assessment of germ cells in testes following administration of aqueous leaf-extract of *Lawsonia inermis* on aluminium-induced oxidative stress in adult Wistar rats

**DOI:** 10.5935/1518-0557.20180080

**Published:** 2019

**Authors:** Toluwase Solomon Olawuyi, Victor Okoliko Ukwenya, Abdul Gafar Akanji Jimoh, Kolade Busuyi Akinola

**Affiliations:** 1 Department of Anatomy, School of Health and Health Technology, Federal University of Technology, Akure (FUTA), Nigeria; 2 Department of Obstrectic & Gynaecology, Faculty of Clinical Sciences, University of Ilorin, Ilorin, Nigeria

**Keywords:** asthenospermia, histomorphometry, teratospermia, spermatogenesis, stereology

## Abstract

**Objectives:**

This study aimed to investigate the 'Cytoprotective effect of *Lawsonia inermis* aqueous leaf-extract on aluminium-induced Oxidative stress in Histomorphometric of the Seminiferous tubule and Stereology of Germ Cells of adult male Wistar rats', assessing its effect on the Histomorphometry of the Seminiferous tubule and Stereology of Germ Cells.

**Methods:**

Thirty-five adult male Wistar rats, weighing between 100-196g, and fifteen mice of the same weight range were used. *Lawsonia inermis* extracts and aluminum chloride (AlCl_3_) were administered for a period of three (3) weeks, with Five (5) rats per group. Group 1 (control), received rat pellets and distilled water. Group 2 received 60mg/kg/d aqueous extract. Group 3 received 0.5mg/kg/d of AlCl_3_. Group 4 received 0.5mg/kg/d of AlCl_3_ and 60mg/kg/d of aqueous extract orally. Group 5 received 0.5mg/kg/d of AlCl_3_ and 75mg/kg/d of aqueous extract orally. Group 6 received 0.5mg/kg/d of AlCl_3_ and 100mg/kg/d of aqueous extract orally. Group 7 received 0.5mg/k/d of AlCl*_3_* and 5mg/Kg/d of ascorbic acid orally. Twenty-four hours after the last administration, the animals were weighed, sedated with chloroform and blood was collected. The testes were removed and weighed.

**Results:**

There were statistically significant changes in the percentage of seminiferous tubular and seminiferous ductal diameter within the experimental animals in all the groups (*p*<0.05). Stereological findings revealed increase in spermatogonia, primary spermatocytes, round Spermatids and elongated spematids, spermatozoa, Sertoli cells population of the control rats while the rats given 0.5mg of aluminum chloride per kg of body weight had the lowest value (*p*<0.05).

**Conclusion:**

In this study, we demonstrated the affected histomorphometry of the seminiferous tubule and stereology of germ cells in testes, where stress impacts were most felt and subsequently translated into drastic reproductive dysfunction and distortion of spermatogenesis.

## INTRODUCTION

Plant-base medicine has been wholly or partially a source of medical therapy (Trivedi, 2006) with an estimated 80% of the world population currently seeking therapeutic solution from herbal medicine as primary health care, and this has gained recognition in several nations of the world, as well as in the World Health Organization (WHO, 2018). For over 9,000 years, Henna, which has the botanical name of *Lawsonia inermis,* has been used as the substance for drawing tattoos on the body. Apart from using *Lawsonia inermis* cosmetically, people use the plant as hair coloring agent in many parts of the world (Sridhar *et al*., 2016). It was reported that all isolated compounds exhibited antioxidant activity comparable to that of ascorbic acid (Mikhaeil *et al*., 2004). Lawsone, 2- hydroxy-1:4 napthaquinone (C10H6O3), is a coloring agent in henna. Besides lawsone, other constituents present are gallic acid, glucose, mannitol, fats, resin (2%), mucilage and traces of an alkaloid. Leaves yield hennatannic acid and an olive oil green resin, soluble in ether and alcohol (Pratibha & Korwar, 1999). Henna contains waxes and coloring matter (Gupta *et al*., 2005). More so, six compounds were identified in *Lawsonia inermis* leaves by GC-MS analysis, and the prevailing compounds were methyl D-Glucopyranoside (51.73%) and lawsone (19.19%) (Hema *et al*., 2010).

Aluminum is known to be the most abundant metal, and the third most common element in the earth's crust (Mohammadirad & Abdollahi, 2011; Onyegeme-Okerenta & Anacletus, 2016). It is found abundantly as trioxosilicate (IV) in rocks and clays. Chemically, it is often found in combination with silicon, fluorine, oxygen, and other earth elements (Sideman & Manor, 1982). It was reported that the oral bioavailability of aluminum could be as low as 0.1%; after absorption, it is distributed into the body of animal and man (Aguilar-Fuentes *et al*., 2008; Walker *et al*., 1990). Aluminum ion is transported in the plasma by the iron binding protein, transferrin and it can enter the brain, placenta and fetus (Macedo & de Sousa, 2008; Fleming & Josh, 1987). Aluminum can be useful in making utensils, cookware, cosmetics, cointainers, and aluminum foil; other primary sources of aluminum include salt, yellow cheese, corn, herbs, teas and spices (Lin *et al*., 1997; Becaria *et al.*, 2002; Sharma *et al*., 2006; Pournourmohammadi *et al*., 2008). It has been reported that aluminum has neurotoxicity effects on the human body, and it is implicated in Alzheimer's disease (Gupta *et al*., 2005; Halliwell & Cutteridge, 1990; Zatta, 2006; Ranjbar *et al.,* 2008). Based on findings by the Agency for Toxic Substances and Disease Registry 'ATSDR', exposure to high levels of aluminum compounds may produce DNA damage (ATSDR, 2008).

According to Ghelberg & Brodas (1981), aluminum is capable of pathologically changing the testes, resulting to testicular atrophy. Toxic effects of aluminum poisoning can cause asthenospermia, hypospermia, teratospermia and reduction in sperm count (Bell & Thomas, 1980). Aluminum has direct effects on the male gonads, consequently, aluminum factory workers experience hypofertility (Lacranjan *et al*., 1975; Bauchinger *et al*., 1976; Abdel-Moneim, 2013).

Functionally, the male reproductive system can be divided into four parts: production of male gametes (spermatozoa) and secretion of testosterone is primarily done by the testes (male gonads), found in the scrotal sac. Direction and deposition of spermatozoa into the female reproductive tract during copulation is done by the ductal system, which is made up of the ductuli efferent, epididymis, ductus (vas) deferens and ejaculatory duct. The connection between the ejaculatory ducts and urethra aids on spermatozoa delivery. Embryologically, each testis descended with the testicular duct system, and neurovascular bundles, as well as a layer of peritoneum, which forms a double layer of mesothelium (tunica vaginalis), that surrounds the scrotum. There is also the tunica albuginea, which splits the testes into several collagenous septa of about 250 testicular lobules. There are one or four convoluted tubes, within the tubules, known as the seminiferous tubules, which is the spermatozoa production chamber. A plexus of channels, called the rete testis, converge beneath the seminiferous tubules. From the rete testis, the ductuli efferents (15 to 20 small ducts) are formed, and they are responsible for the transmission of spermatozoa to the ductus deferens (the epididymis).

This study aimed to investigate the 'cytoprotective effect of *Lawsonia inermis* aqueous leaf-extract on aluminum-induced oxidative stress on the histomorphometry of the seminiferous tubules and the stereology of germ cells of adult male Wistar rats'.

## MATERIALS AND METHODS

Aluminum chloride and ascorbic acid were bought in the Mich-Deson Hospital equipment store, Upper Taiwo, Ilorin. The histological staining was done in Anatomical-pathology Department, University Teaching Hospital Ilorin, Nigeria.

### Preparation of Extracts 

The plant was obtained from Isanlu-Isin in Kwara State, Nigeria and identified professionally by the herbarium number UPH/P/114, by the taxonomist in the Department of Plant Science and Biotechnology - University of Port-Harcourt, Rivers State, Nigeria. The Research Ethics Committee of the same institution approved this study on the 25^th^ of February, 2016, under reference number UPH/CEREMAD/REC/04. The plant leaves were washed with water, cut into pieces, dried in a cool environment. The dried plant leaves were pulverized into coarse powder in a grinding machine. The filtrate was concentrated using a rotary evaporator (Buchi), and further concentrated to dryness at 50ºc in an electric oven (GallenKamp). After drying, it was stored in the refrigerator at 4ºc until needed for use.

### Acute Toxicity Test (LD*_50_*)

Fifteen mice were used to conduct the above test, to determine the safe dosages and lethal dosage. They were grouped into five (5), with three (3) mice per group. The acute toxicity of the *Lawsonia inermis* extract was assessed by the LD*_50_* calculation, using a limit dose test at a limit dose of 1000mg/kg body weight of the extract after oral administration in mice (three animals per group) (OECD-OCDE 425 Guide). Using the oral route, the animals showed dose-dependent signs of toxicity, ranging from lack of appetite, depression, immobility and respiratory distress to death. LD*_50_* for *Lawsonia inermis* extract is 0.75g while the safe dose is 0.1g/Kg b.w.

### Determination of the Extract Dosage to Administer 

The choice of dosage based on the Dosage-Acute-Toxicity test (LD*_50_*) above, the safe dose of *Lawsonia inermisis* is 0.1g/Kg or 100mg/Kg of body weight. The highest dose is 100mg/Kg, the medium dose is 75mg/Kg and the lowest dose is 60mg/Kg.

### Animal Breeding

Thirty-five adult male Wistar rats and fifteen mice were used, with an average weight of 100-196g. The rats were housed in cages (made of wood, wire gauze and net) under natural light and dark cycles, at room temperature in the animal house of the Faculty of Basic Medical Sciences, University of Ilorin. The floor of the cages was made of wood to make it comfortable for the rats, and it was covered with sawdust to provide a soft floor for the rats and to make cleaning of the cage convenient when littered. They were fed with rat pellets purchased from stores approved by the University of Ilorin, and water was given *ad libitum*. They were grouped and left to acclimatize for 2 weeks before the study commenced.

### Grouping

The total numbers of animals was thirty-five. They were grouped into one (1) control and six (6) experimental groups with consideration towards size variation. Using a feeding tube (size-6), distilled water, and *Lawsonia inermis* extracts were administered to the control and treated animals respectively for a period of three (3) weeks.

Group 1 (control): (n=5): Given rat pellets and distilled water.

Group 2: (n=5): Given 60mg/kg/d extract of *Lawsonia inermis* and pellets.

Group 3: (n=5): Given0.5mg/kg/d of aluminum chloride in distilled water and pellets.

Group 4: (n=5): Given 0.5mg/kg/d of aluminum chloride and low dose 60mg/kg/d of *Lawsonia inermis* in distilled water orally. 

Group 5: (n=5): Given 0.5mg/kg/d of aluminum chloride and medium dose 75mg/kg/d of *Lawsonia inermis* orally.

Group 6: (n=5): Given 0.5mg/kg/d of aluminum chloride and high dose 100mg/kg/d of *Lawsonia inermis* in distilled water orally.

Group 7: (n=5): Given 0.5mg/k/d of aluminum chloride and 5mg/Kg/d Ascorbic acid in distilled water orally.

### Animal Slaughter and Sample Collection 

Twenty-four hours after the last administration, the animals were weighed and thereafter slaughtered by the use of chloroform as a sedative. Their abdominal cavities were opened by a midline abdominal incision and the reproductive organs (testes) were removed.

### Histomorphometric (Stereological) Evaluation of the Testes

Histomorphometric analysis using 'Imagej®' (an open source image processing software, designed for scientific multidimensional images): the science that studies the reaction between antigen and antibodies in serum. Sections of 3µm were stained with PAS-H, and H & E was used for stereological studies. Histomorphometric data was collected with the aid of a Leica (DM 750) digital microscope (Leica Microsystems, Switzerland) and connected to a computer (Plates A and B).

**Plate A:**
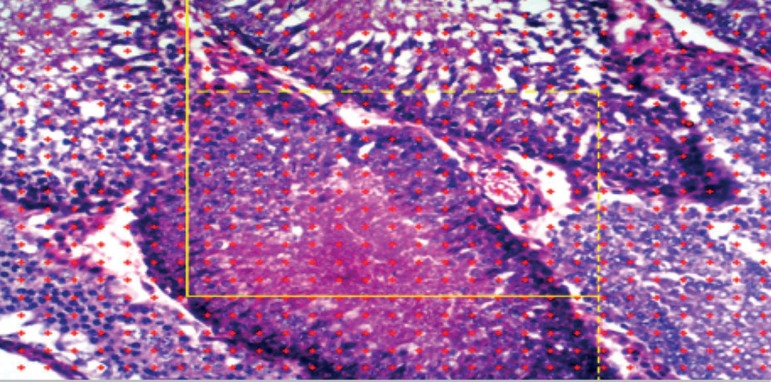
The percentage of seminiferous tubules, Leydig cells, Non-Leydig extral cellular cells through the sample window of image. The number of dots inside the box fell on the seminiferous tubules was calculated in percentage likewise the dot fell on Leydig and non-Leydig extral cellular cells

**Plate B:**
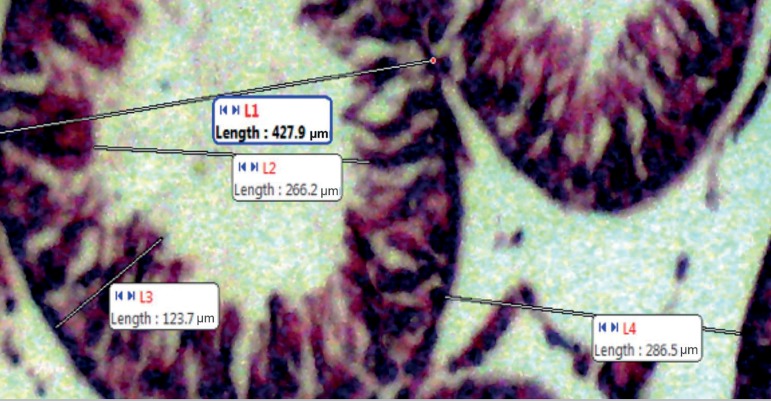
The Seminiferous Luminal diameter ‘L1’, Seminiferous ductal diameter ‘L2’, Seminiferous Epithelia height ‘L3’, Interstitial space diameter ‘L4’ in microns

### Stereological Determination of Germ and Somatic Cells in Testis

All germ cell nuclei and Sertoli cell nucleoli present at *stage* VII of the cycle were counted in 10 circular or nearly circular seminiferous tubule cross sections chosen at random, for each animal. These counts were corrected for section thickness and nucleus or nucleolus diameter according to Abercrombie (1946) and modified by Amann (1962).

## RESULTS

**Figure 1:**
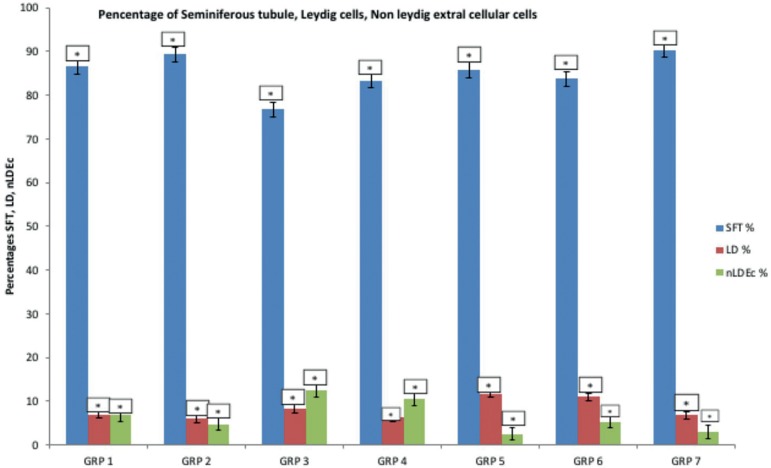
Variation in the percentages of seminiferous tubules, Leydig cells, Non-Leydig extral cellular cells

There were statistically significant changes in the percentage of seminiferous tubular differences of the experimental animals in all the groups (*p*<0.05). The mean and standard error of mean (sem) for the percentage of seminiferous tubule in group 7 (given ascorbic acid with aluminium chloride) and group 2 (given the extract alone) were the highest (90.3±2.3%), while the least was group 3 (given aluminium chloride alone), (76.8±5.8%) (Figure 1). There were no statistically significant changes in the percentage of Leydig cells and non-Leydig extra cellular cell differences of the testes in all the groups (*p*>0.05). Figure 1 shows the mean and standard error of mean for Leydig cells in Group 5 (given a medium dose extract with aluminum chloride) was the highest (11.8±2.3%), followed by group 6 (given high dose of extract with aluminum) (11.0±1.7%), while group 2 (extract alone) was the least (6.0±0.71%). According to Figure 1, the mean and standard error of mean for non-Leydig extral cellular cells in Group 3 (Aluminum chloride) was the highest (12.5±6.1%), while group 5 (medium dose extract with aluminum chloride) was the least (2.5±0.29%).

**Figure 2:**
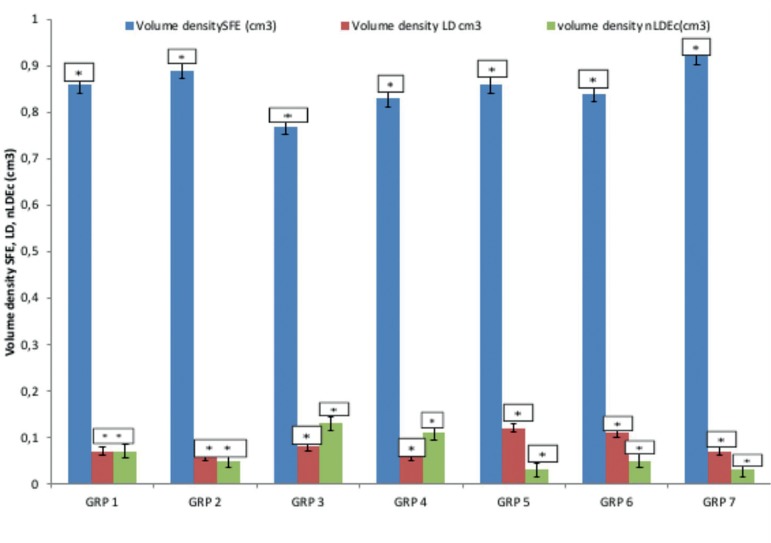
Variation in the volume densities of seminiferous tubules, Leydig cells, Non-Leydig extral cellular cells

There were statistically significant changes in the volume density of seminiferous tubules difference of the experimental animals in all the groups (*p*<0.05). The mean (±sem) for the volume density of Seminiferous tubule in group 7 (given ascorbic acid with aluminium chloride) (0.92±0.03cm^3^) was the highest, followed by group 2 (given Extract alone) (0.89±0.01cm^3^), while the least was group 3 (given aluminium chloride alone), (0.77±0.06cm^3^) (Figure 2).There were no statistically significant changes in the Volume density of Leydig cells and non-Leydig extral cellular cells differences in all the groups (*p*>0.05). Figure 2 shows the mean (±sem) for Leydig cells in Group 5 (given medium dose extract with aluminum chloride) was higher than other groups (0.12±0.022cm^3^), followed by group 6 (given high dose extract with aluminum) (0.11±0.017cm^3^), while group 2 (extract alone) and group 4 (low dose extract with aluminum chloride) were the least (0.06±0.007cm^3^). According to Figure 2, the mean (±sem) for non-Leydig extral cellular cells in Group 3 (Aluminum chloride) was the highest (0.13±0.061cm^3^) while group 5 (medium dose extract with aluminum chloride) and group 7 (ascorbic acid with aluminum chloride) were the lowest (0.03±0.003cm^3^).

The mean seminiferous ductal diameter within the experimental animals in all the groups was significantly different (*p*<0.05). From Table 1, it was found that group 7 (ascorbic with aluminum chloride) had the highest mean value (80.6±8.0µm), followed by group 5 (medium-dose extract with aluminum chloride) (66.1±10.8µm), while the group with the lowest value was group 4 (lowest dose extract with aluminum chloride) (39.0±2.4µm). There were markedly significant statistical differences in seminiferous luminal diameter in the experimental animals testes in all the groups (*p*<0.05). The mean (±sem) for the seminiferous luminal diameter in group 4 (given the lowest dose of *lawsonia inermis* with aluminum chloride) (9.4±0.5µm), followed by group 2 (*lawsonia inermis* alone) (3.8±0.7µm) (Table 1); while group 7 (ascorbic acid with aluminum chloride) had the least mean value (1.8±0.6µm) (Table 1).

**Table 1:** The Distribution of Mean and standard error of mean (sem) for the seminiferous ductal, Luminal diameters, Epithelia height, interstitial space diameter in micron.

GROUPS	S.ductal diameter (µm)	S. Luminal diameter (µm)	S.Epithelia height (µm)	Interstial space Diameter (µm)
**GRP 1**	55.4±1.8	3.4±1.3	12.1±2.4	2.5±0.6
**GRP 2**	41.9±1.2	3.8±0.7	10.3±0.7	3.0±0.7
**GRP 3**	61.8±5.9	3.6±1.7	2.9± .7	24.8±5.5
**GRP 4**	39.0±2.4	9.4±0.5	2.9±0.3	6.4±1.6
**GRP 5**	66.1±10.8	3.0±0.2	8.6±0.8	4.6±0.5
**GRP 6**	58.1±3.6	3.7±1.1	10.8±0.5	7.4±0.2
**GRP 7**	80.6±8.0	1.8±0.6	12.9±1.1	6.5±0.2

There were statistically significant differences in the seminiferous epithelia height of the experimental animals in all the groups (*p*>0.05). The mean value (±sem) for the seminiferous epithelia height in group 7 (given ascorbic acid with aluminium chloride) (12.9±1.1µm), followed by group 1 (control) (12.1± .4µm) (Table 1); while group 3 (aluminium chloride alone) and group 4 (given lowest dose of *lawsonia inermis* with aluminium chloride) had the lowest mean of 2.9±0.7µm (Table 1). Lastly, the interstitial space diameters of the experimental animals in all the groups were significantly different (*p*<0.05). Table 1 shows that the mean (±sem) for the interstitial space diameter in group 3 (given aluminium chloride alone) was 24.8±5.5µm followed by group 6 (given highest dose *lawsonia inermis* with aluminium chloride) 7.4±0.2µm (Table 1); while group 1 (control) had the lowest mean (2.5±0.6µm) (Table 1). 

The germ cell count was Spermatogonia, Primary Spermatocyte, Secondary Spermatocyte, Round Spermatid, Elongated Spermatid, Spermatozoa and Sertoli Cells. According to Table 2, the control rats had the highest spermatogonia standard error of mean, that is: (46.1 1.9)x10*^6^*/cm^3^, while the rats given 0.5mg of aluminum chloride per kg of body weight had the lowest standard error of mean: (14±0.7)x10*^6^*cm^3^. Stereological determination of primary spermatocytes showed that the control rats had the highest standard error of mean, that is: (48.4±2.2)x10*^6^*/cm^3^, while the rats given 0.5mg of aluminum chloride per kg of body weight and medium dose of 75mg/kg of *Lawsonia inermis* had the lowest standard error of mean: 20.1±0.8)x10*^6^*/cm^3^. Secondary spermatocytes were the highest in the rats given 60mg/kg of *Lawsonia inermis*, (83±2.6)x10*^6^*/cm^3^, and lowest in the rats given 0.5mg of aluminum chloride per kg of body weight, the standard error of mean value was: 18.4±0.7x10*^6^*/cm^3^.

**Table 2:** The Distribution of Mean and standard error of mean (sem) for the spermatogonia, Primary Spermatocyte, Secondary Spermatocyte, Round Spermatid, Elongated Spermatid, Spermatozoa and Sertoli Cells in micron per cube

GROUP	Spermatogonia X10*^6^*/cm^3^	Primary Spermatocyte X10*^6^*/cm^3^	Secondary Spermatocyte X10*^6^*/cm^3^	Round Spermatid X10*^6^*/cm^3^	Elongated Spermatid X10*^6^*/cm^3^	Spermatozoa X10*^6^*/cm^3^	Sertoli Cells X10*^6^*/cm^3^
**Grp 1**	46.1±1.9	48.4±2.2	59.6±2.4	22.5±0.9	58.5±1.3	76.5±1.4	13.5±0.3
**Grp 2**	43.0±1.2	40.0±1.8	83.0±2.6	31.0±0.5	91.0±2.2	102.0±2.1	8.0±0.1
**Grp 3**	14.0±0.7	28.9±1.3	18.4±0.7	14.0±0.4	7.9±0.2	4.4±0.1	4.4±0.1
**Grp 4**	39.1±1.2	28.9±0.9	30.6±1.8	14.0±0.3	29.8±0.9	33.3±1.0	7.9±0.2
**Grp 5**	35.0±1.4	20.1±0.8	41.1±1.8	40.3±1.3	35.0±0.8	43.8±1.2	10.5±0.1
**Grp 6**	20.9±1.1	23.8±1.1	53.2±1.7	59.9±1.3	53.2±1.1	68.4±1.4	8.6±0.2
**Grp 7**	24.8±0.8	21.8±0.9	36.0±0.8	40.5±1.2	39.7±0.8	67.5±1.7	6.8±0.1

Stereological determination of round spermatids, and elongated spematids were the highest for animals given 0.5mg of aluminum chloride per kg of body weight and high dose of 100mg/kg of *Lawsonia inermis* (59.9±1.3)x10*^6^*/cm^3^; also, the rats given 60mg/kg of *Lawsonia inermis was* 91±2.2x10*^6^*cm^3^. The lowest standard error of means for both cells: (14±0.4)x10*^6^*cm^3^ in the rats given 0.5mg of aluminum chloride per kg of body weight with a low dose 60mg/kg of *Lawsonia inermis and* 0.5mg of aluminum chloride per kg of body weight alone, while elongated spermatids were: (7.9±0.2)x10*^6^*/cm^3^, animals given 0.5mg of aluminum chloride per kg of body weight.

Stereological determination of spermatozoa shows that the rats given 60mg/kg of *Lawsonia inermis* alone had the highest standard error of mean, that is: (102±2.1)x10*^6^*cm^3^ while the rats given 0.5mg of aluminum chloride per kg of body weight had the lowest mean (4.4±0.1)x10*^6^*/cm^3^. Lastly, according to table 2, the control rats had the highest Sertoli cell standard error of mean, that is: (13.5±0.3)x10*^6^*/cm^3^ while the rats given 0.5mg of aluminum chloride per kg of body weight had the lowest standard error of mean of (4.4±0.1)x10*^6^*/cm^3^.

## DISCUSSION

The seminiferous tubule of the rats given ascorbic acid with aluminium chloride and rats given extract alone were the widest, which is probably due to fluid secretion by Sertoli cells to facilitate spermiation, while the least were the rats given aluminium chloride alone (Figure 1). In agreement with Xiao *et al.* (2014), this suggests reduced Sertoli fluid secretion and may represent the initial *stage* of spermiation failure. However, it must be understood that reduced Sertoli secretion certainly reflect dysregulation at a molecular level, possible molecular players in spermiation identified so far include actin bundling proteins such as epidermal growth factor receptor pathway substrate 8 (Eps8) and actin cross-linking and binding protein paladin. There were no statistically significant changes in the percentage of Leydig cells and non-Leydig extral cellular cells differences of the testis in all the groups (*p*>0.05). Figure 1 shows that Leydig cells of the rats given medium dose extract with aluminum chloride, were the widest, followed by the rats given high dose extract with aluminum while rats with extract alone were the least. According to Figure 1, the rats that were given aluminum chloride alone had the highest values of non-Leydig extral cellular cells while the rats with medium dose extract with aluminum chloride were the lowest.

There were statistically significant changes in the volume density of seminiferous tubules of the experimental animals in all the groups (*p*<0.05). The volume density of seminiferous tubule in the rats given ascorbic acid with aluminium chloride were the highest followed by the rats given the extract alone, while the lowest were associated with the rats given aluminium chloride alone (Figure 2). This result is in agreement with Russell *et al.* (1990), as they documented that seminiferous tubules were used to locate the occurrence of spermatogenesis, in which the spermatogonia mature into sperm.

There were no statistically significant changes in the volume density of Leydig cells and non-Leydig cells in all the groups (*p*>0.05). Figure 2 shows that the number of Leydig cells of the rats given a medium dose of 75mg/kg of *Lawsonia inermis* aqueous leaf-extract with aluminum chloride was the highest, followed by the rats given a high dose of 100mg/kg of *Lawsonia inermis* aqueous leaf-extract with aluminum chloride, while the rats given 60mg/kg of *Lawsonia inermis* aqueous leaf-extract alone and the rats given a low dose of 60mg/kg of *Lawsonia inermis* aqueous leaf-extract with aluminum chloride were the lowest. Increase in the volume density of Leydig cells is very important; Franca & Russell (1998) reported that the production of androgens by Leydig cells is the factor for the initiation of secondary sexual characteristics and the maintenance of spermatogenesis. An increase in the cytoplasmic and nuclear volume, and consequently increase in the individual cellular volume could be associated with increased availability of testosterone.

According to Figure 2, the non-Leydig extral cellular cells rats given aluminum chloride were the highest while rats given medium dose extract with aluminum chloride and rats given ascorbic acid with aluminum chloride were the lowest. Franca & Russell (1998) reported that Leydig cells are found in the intertubular compartment such as neurovascular structures and connective tissue fibers, macrophages, and mast cells.

Statistical differences were found in the seminiferous ductal diameters among the experimental animals in all the groups (*p*<0.05). Table 1 shows that rats given ascorbic acid with aluminium chloride had the highest value followed by the rats given medium doses of extract with aluminum chloride, while the group with the lowest value was given the lowest dose of extract with aluminum chloride. Statistical differences were markedly significant in seminiferous luminal diameters in the experimental animals testes in all the groups (*p*<0.05). The seminiferous luminal diameter of the rats given the lowest dose of 60mg/kg of *Lawsonia inermis* with aluminium chloride, followed by the rats given *lawsonia inermis* alone (Table 1); while the rats given ascorbic acid with aluminium chloride had the lowest value (Table 1). According to Santamarina & Reece (1957), the process of lumination seems to be governed by two phenomena. First, the dying germ cells always leave variably sized vacuoles in the center of the cords, which coalesce to form larger spaces that may represent the primary elements of lumination. On the other hand, the high rate of division of germ cells in the premature stage and the concomitant access of these cells to the basal lamina may initiate lengthening of this membrane with subsequent increase in its external diameter, and to open the center of seminiferous cords that means lumination.

There were statistically significant differences in the seminiferous epithelia height of the experimental animals in all the groups (*p*>0.05). The seminiferous epithelia height in rats given ascorbic acid with aluminum chloride followed by the control group had the highest value (Table 1); while the rats given aluminum chloride alone and the rats given the lowest dose of *lawsonia inermis* with aluminum chloride had the lowest value (Table 1). The seminiferous epithelia height is under the influence of hormonal regulation of the male reproductive system; the spermatogenic epithelium is induced by follicle stimulating hormone (FSH). Russell *et al*. (1990) reported that increase in the height of the seminiferous tubule epithelium could increase the sperm production process.

Lastly, statistically significant differences were found in the interstitial space diameter within the experimental animals in all the groups (*p*<0.05). Table 1 shows the interstitial space diameter of the rats given aluminium chloride alone, followed by the rats given the highest dose of *lawsonia inermis* with aluminium chloride (Table 1); while the control group had the lowest value (Table 1).

Different germ cells were found in the seminiferous tubules (Table 2). Spermatogonia were irregular or oval and showed variable contact with the basement membrane. Their nuclei were large, oval-shaped and showing inconstant nucleoli together with coarse chromatin particles. Primary spermatocytes were the second layer of germ cells; they were oval or rounded and possessing rounded centrally located nuclei with coarse chromatin aggregations and less defined nuclear membrane. The secondary spermatocytes were found only in some tubules and were smaller than the primary ones. Their nuclei were also smaller than those of primary spermatocytes were, but had distinct nuclear membrane and few chromatin particles. Spermatids (round and elongated) at different stages of transformation were found in some seminiferous tubules. In addition, spermatozoa were located in the luminal region of the seminiferous tubules.

This current finding revealed an increase in the spermatogonia population in control rats; the rats with lawsonia inermis aqueous extract alone; while the rats given 0.5mg of aluminum chloride per kg of body weight had the lowest population of spermatogonia (Table 2), followed this. The interpretation of this spermatogonia increase in both groups 1 and 2 can be seen in Table 2. The follicular stimulating hormone, luteinizing hormone and testosterone were the highest, and these have positive influence on the spermatogonia proliferation.

Stereological findings in this study revealed increase in the primary spermatocyte population of the control rats, while the rats given 0.5mg of aluminum chloride per kg of body weight and a medium dose of 75mg/kg of *Lawsonia inermis* had the lowest population. Meanwhile, the secondary spermatocytes of the rats given 60mg/kg of *Lawsonia inermis,* were more populous than the other group, and least were the rats given 0.5mg of aluminum chloride per kg of body weight. Testosterone partly supports spermatocyte maturation; Table 2 shows high levels of testosterone in groups 1 and 2 compared with the other groups; hence, there were increased spermatocytes. Equally, Table 2 shows increases in seminiferous tubular percentage in both groups 1 and 2, this provides a large environment for the proliferation of primary and secondary spermatocytes.

Stereological determination of round spermatids and elongated spematids was the highest for animals given 0.5mg of aluminum chloride per kg of body weight and a high dose of 100mg/kg of *Lawsonia inermis* also, the rats given 60mg/kg of *Lawsonia inermis*. The rats given 0.5mg of aluminum chloride per kg of body weight with a low dose of 60mg/kg of *Lawsonia inermis and* 0.5mg of aluminum chloride per kg of body weight alone were having the lowest population of spermatids.

The impact of elevated GnRH on the round spermatids is felt on groups 2 and 6 in the rats given 0.5mg of aluminum chloride per kg of body weight and a high dose of 100mg/kg of *Lawsonia inermis* also, the rats given 60mg/kg of *Lawsonia inermis*, though more severe in the rats given 0.5mg of aluminum chloride per kg of body weight with a low dose of 60mg/kg of *Lawsonia inermis and* 0.5mg of aluminum chloride per kg of body weight alone. It is possible that spermatogenesis is more susceptible to the myriad of perturbations caused by elevated GnRH and testosterone levels, it is clear that the seemly negligible reduction in germ cell populations at proliferative and meiotic phases contribute to the significant reduction witnessed at the differentiation phase. The loss of germ cells during the proliferative and spermatogenic phases is indeed statistically significant, the resultant effect of such loss combines with losses at spermatogenic differentiation phase to produce a dramatic reduction in spermatozoa output. Putting all these observational data together, it becomes clear that oxidative stress, as an intangible testicular toxicant, produces a multi-phasic target against spermatogenesis, causing insignificant effects on the proliferative phase, which compromises the number of primary spermatocytes derivable from mitosis, and then separately causes meiotic deficiency, thereby further compromising the number of spermatocyte entering the differentiation phase - the *stage* at which more structural damages are separately done to spermatids, and resulting low spermatozoa.

Stereological determination of Sertoli cells in this current study, according to table 2 the control rats have the highest number of Sertoli cells while the rats given 0.5mg of aluminum chloride per kg of body weight had the lowest value. According to Xiong *et al*. (2009) the role of residual bodies in Sertoli cell metabolism may provide some understanding of its implication. The residual bodies through the tubular current may weaken Sertoli energetics by reducing its adenosine triphosphate (ATP) formation capacity. Sertoli cells depend predominantly on lipid β-oxidation for ATP production and both apoptotic spermatogenic cell and residual bodies undergo phagocytosis by Sertoli to become fat droplets, which are then used as sources of energy for ATP production. When Sertoli cells are denied this priceless source of metabolic fuel, through the efflux of residual bodies as seen suspended in the lumen of tubules in the rats given 0.5mg of aluminum chloride per kg of body weight, the potential danger is beyond conjecture and includes compromised Sertoli ability to synthetize proteins and molecules needed to maintain their relationship with and nourish germ cells. The rats given 0.5mg of aluminum chloride per kg of body weight will be more detrimental to spermatogenesis than when residual bodies are considered as excess cytoplasmic luggage meant for disposal either by phagocytosis or lumen effluxion.

In all probability, the failure of a large number of residual bodies to be transported back towards the basement membrane may represent Sertoli cell phygocytotic dysfunction. Whatever the cause, this abnormality can go into a vicious cycle where Sertoli cells have less and less available oxidisable fuel for its critical function of maintenance of appropriate anatomical relationship with and nourishing germ cells at different *stage* of spermatogenesis. Sertoli cells assists in mechanically and nutritionally supporting the spermatogenic cells and the secretion of two hormones; inhibin and activin, provide positive and negative feedback for regulating FSH secretion from the pituitary (Slomianka, 2009). It was obvious that oxidative stress induced by aluminum poison decrease FSH secretion, while the concomitant addition of aluminum and *Lawsonia inermis* ameliorated this negative effect.

## CONCLUSION 

It was obvious that the oxidative stress negatively affects male reproduction. This study has demonstrated the effects on histomorphometry of the seminiferous tubule and stereology of germ cells in testes where stress impacts were most felt and subsequently translated into drastic reproductive dysfunction and distortion of spermatogenesis.

## References

[r1] Abdel-Moneim A. (2013). Effects of taurine against histomorphological and ultrastructural
changes in the testes of mice exposed to aluminium chloride. Arh Hig Rada Toksikol.

[r2] Abercrombie M. (1946). Estimation of nuclear populations from microtome
sections. Anat Rec.

[r3] Aguilar-Fuentes J, Fregoso M, Herrera M, Reynaud E, Braun C, Egly JM, Zurita M (2008). p8/TTDA overexpression enhances UV-irradiation resistance and
suppresses TFIIH mutations in a Drosophila trichothiodystrophy
model. PLoS Genet.

[r4] Amann RP (1962). Reproductive capacity of dairy bulls. III. The effect of
ejaculation frequency, unilateral vasectomy, and age on
spermatogenesis. Am J Anat.

[r5] ATSDR, Agency for Toxic Substances and Disease Registry (2008). Toxicological profile for Aluminum Department of Health and Human
Services.

[r6] Bauchinger M, Schmid E, Einbrodt HJ, Dresp J (1976). Chromosome aberration in lymphocytes after occupational exposure
to lead and cadmium. Mutat Res.

[r7] Becaria A, Campbell A, Bondy SC (2002). Aluminium as a toxicant. Toxicol Ind Health.

[r8] Bell JU, Thomas JA, Singhal RL, Thomas JA (1980). Effects of lead on mammalian reproduction. Lead toxicity.

[r9] Fleming J, Josh JG (1987). Ferritin: Isolation of aluminium-ferrittin complex from
brain. Proc Natl Acad Sci U S A.

[r10] Franca LR, Russell LD, Martinez-Garcia F, Regadera J (1998). The testis of domestic mammals. Male Reproduction: A Multidisciplinary Overview.

[r11] Ghelberg NW, Brodas E (1981). Lead induced experimental lesions of the testes and their
treatment. J Appl Toxicol.

[r12] Gupta VB, Anitha G, Hegda ML, Zecca L, Garruto RM, Ravid R, Shankar SK, Stein R, Shanmugavelu P, Jagannatha Rao KS (2005). Aluminium in Alzheimer's disease: Are we still at a
crossroad?. Cell Mol Life Sci.

[r13] Halliwell B, Cutteridge JM (1990). Role of free radical and catalytic metal ions in human disease:
an overwiew. Methods Enzymol.

[r14] Hema R, Kumaravel S, Gomathi N, Sivasubramaniam C (2010). Gas Chromatography - Mass Spectroscopic analysis of Lawsonia
Inermis leaves. N Y Sci J.

[r15] Lacranjan I, Popescu HI, Gavenescu O, Klepsch I, Serbănescu M (1975). Reproductive ability of workmen occupationally exposed to
lead. Arch Environ Health.

[r16] Lin JL, Yang YJ, Yang SS, Leu ML (1997). Aluminum utensile contributes to aluminum accumulation in
patients with renal disease. Am J Kidney Dis.

[r17] Macedo MF, de Sousa M (2008). Transferrin and the transferrin receptor: of magic bullets and
other concerns. Inflamm Allergy Drug Targets.

[r18] Mikhaeil BR, Badria FA, Maatooq GT, Amer MM (2004). Antioxidant and immunomodulatory constituents of henna
leaves. Z Naturforsch C.

[r19] Mohammadirad A, Abdollahi M (2011). A systematic review on oxidant/ Antioxidant imbalance in
Aluminium toxicity. Int J Pharmacol.

[r20] Onyegeme-Okerenta BM, Anacletus FC (2016). Evaluation of aluminium toxicity and the ameliorative effect of
some selected antioxidants on fecundity of matured male wister rats (rattus
rattus). Eur J Adv Res Biol Life Sci.

[r21] Pournourmohammadi S, Khazaeli P, Eslamizad A, Tajvar A, Mohammadirad A, Abdollahi M (2008). Study on the oxidative stress status among cement plant
workers. Hum Exp Toxicol.

[r22] Pratibha G, Korwar GR (1999). Estimation of lawsone in Henna (Lawsonia inermis). J Med Aromat Plant Sci.

[r23] Ranjbar A, Khani-Jazani R, Sedighi A, Jalali-Mashayekhi F, Ghazi-Khansari M, Abdollahi M (2008). Alteration of body total antioxidant capacity and thiol molecules
in human Chronic exposure to aluminum. Toxicol Environ Chem.

[r24] Russell L, Ettlin RA, Hikim SAP, Clegg EJ (1990). Histological and Histopathological Evaluation of the
Testis.

[r25] Santamarina E, Reece RP (1957). Normal development of the germinal epithelium and seminiferous
tubules in the bull. Am J Vet Res.

[r26] Sharma N, He Q, Sharma RP (2006). Amelioration of fumonisin B1 hepatotoxicity in mice by depletion
of T cells with anti-Thy-1.2. Toxicology.

[r27] Sideman S, Manor D (1982). The dialysis dementia syndrome and aluminum
intoxication. Nephron.

[r28] Slomianka L (2009). Blue Histology - Male Reproductive System. School of Anatomy and
Human Biology. The University of Western Australia.

[r30] Sridhar VR, Jayakumar P, Arun S, Jaikumar S (2016). Sedative effect of Lawsonia inermis root extract on
Phenobarbitone induced sleeping time in mice. Eur J Biol Biochem.

[r31] Trivedi PC (2006). Medicinal plants: Traditional Knowledge.

[r32] Walker JA, Sharman RA, Cody RP (1990). The effect of oral bases on enteral aluminum
absorption. Arch Intern Med.

[r33] WHO - World Health Organization (2018). Traditional, complementary and integrative medicine.

[r34] Xiao X, Mruk DD, Wong CK, Cheng CY (2014). Germ cell transport across the seminiferous epithelium during
spermatogenesis. Physiology (Bethesda).

[r35] Xiong W, Wang H, Wu H, Chen Y, Han D (2009). Apoptotic spermatogenic cells can be energy sources for Sertoli
cells. Reproduction.

[r36] Zatta P. (2006). Aluminium and Alzheimer's disease: A Vexata Questio between
uncertain data and a lot of imagination. J Alzheimers Dis.

